# Advances and Challenges in Two-Dimensional Organic–Inorganic Hybrid Perovskites Toward High-Performance Light-Emitting Diodes

**DOI:** 10.1007/s40820-021-00685-5

**Published:** 2021-08-02

**Authors:** Miao Ren, Sheng Cao, Jialong Zhao, Bingsuo Zou, Ruosheng Zeng

**Affiliations:** School of Physical Science and Technology, MOE Key Laboratory of New Processing Technology for Non-Ferrous Metals and Materials, Guangxi Key Laboratory of Processing for Non-Ferrous Metals and Featured Materials, Guangxi University, Nanning, 530004 People’s Republic of China

**Keywords:** Two-dimension, Perovskite, Light-emitting diodes, Optical properties

## Abstract

Two-dimensional (2D) perovskites are known as one of the most promising luminescent materials due to their structural diversity and outstanding optoelectronic properties. Compared with 3D perovskites, 2D perovskites have natural quantum well structures, large exciton binding energy (*E*_*b*_) and outstanding thermal stability, which shows great potential in the next-generation displays and solid-state lighting. In this review, the fundamental structure, photophysical and electrical properties of 2D perovskite films were illustrated systematically. Based on the advantages of 2D perovskites, such as special energy funnel process, ultra-fast energy transfer, dense film and low efficiency roll-off, the remarkable achievements of 2D perovskite light-emitting diodes (PeLEDs) are summarized, and exciting challenges of 2D perovskite are also discussed. An outlook on further improving the efficiency of pure-blue PeLEDs, enhancing the operational stability of PeLEDs and reducing the toxicity to push this field forward was also provided. This review provides an overview of the recent developments of 2D perovskite materials and LED applications, and outlining challenges for achieving the high-performance devices.

## Highlights

- The fundamental structure, photophysical and electrical properties of 2D perovskite films were illustrated systematically.
- The advantages and challenges of 2D perovskite light-emitting diodes (PeLED) have been also discussed, which may benefit the audient to get insight into the 2D perovskite materials as well as the resultant LED devices.
- An outlook on further improving the efficiency of pure-blue PeLEDs, enhancing the operational stability of PeLEDs and reducing the toxicity to push this field forward was also provided.

## Introduction

Perovskite materials have attracted extensive attention due to their interesting properties such as photoluminescence, electroluminescence, high carrier mobility, large optical absorption coefficient and good nonlinear optical properties [1–10]. The chemical composition of traditional perovskite is CaTiO_3_, which belongs to orthorhombic system. The origin of perovskite dates back to 1839 when Gustav Rose, a German mineralogist, discovered perovskite rock samples in the Ural Mountains and named after L. A. Perovski, a Russian geologist. After that, materials with similar structure to CaTiO_3_ are called perovskite. The formula of three-dimensional (3D) halide perovskite is ABX_3_ (A = MA^+^, FA^+^, Cs^+^, Rb^+^, K^+^; B = Pb^2+^, Cu^2+^, Sn^2+^; X = Cl^−^, Br^−^, I^−^) [11–21].

The preparation of CsPbX_3_ was successfully reported in 1893 [22]. However, it was not until the 1950s that perovskite crystal structure and properties were discovered. In 1978, the crystal structure and properties of MAPbX_3_ were synthesized and determined by Weber et al. [23]. Subsequently, Mitzi et al. further studied the unique optoelectronic properties of three-dimensional (3D) perovskites [24, 25]. However, until 2009, perovskite materials broadly attracted researchers’ interest. Miyasaka reported for the first time that MAPbBr_3_ and MAPbI_3_ sensitized TiO_2_ to achieve visible light conversion in photoelectrochemical cells [26]. Beyond solar cells, perovskite materials were also used in LEDs. In 2014, Tan et al. [27] realized near-infrared, green and red room temperature electroluminescence by adjusting the halide compositions in perovskites, which used 3D methylammonium lead halide perovskites as the emissive layer. However, the focus has gradually shifted to low-dimensional perovskite films. Different from three-dimensional perovskites, there is at least one large organic cation in the low-dimensional perovskites, which cannot match the cubic center. Interestingly, 3D, 2D, 1D and 0D perovskites can be formed by different bonding methods of octahedral [BX_6_]^4−^. At the same time, 2D perovskite materials have unique physical and chemical properties due to the influences of quantum confinement and dielectric confinement effect [28]. In addition, the existence of organic cations greatly improves the stability of perovskites [29]. Therefore, 2D perovskites have become star materials used in many fields, including LEDs [30, 31], solar cells [32–35], photodetectors [36, 37], lasers [38, 39] and sensors [40].

In the early stage, the LEDs with 2D perovskites as the emissive layer can only observe the electroluminescence at low temperature. In recent years, a quasi-2D perovskite structure has been proposed by introducing organic cations into 3D perovskites, which can assemble multiple quantum wells. The existence of quantum wells not only benefits the formation of exciton, but also increases the difficulty of exciton separation. This makes quasi-2D perovskite materials promising for high-performance LEDs. Sargent et al. [41] realized the photoexcitation funneling to the lowest bandgap emitter in the mixture by dimensional modulation. In 2016, Huang et al. [42] reported a multi quantum well-based PeLED, showing a very high external quantum efficiency (EQE) of 11.7%. Recently, Lee et al. [43] prepared high-quality quasi-2D perovskite films by a simple and novel encapsulation growth method and obtained high-efficiency sky-blue LEDs with maximum EQE of 12.8%. Di et al. [44] reported near-infrared LEDs with quasi-2D and 3D perovskites and insulating polymers as emissive layers. The EQE was as high as 20.1%. Su et al. [45] proposed a co-interlayer engineering strategy for the preparation of quasi-2D perovskite materials, which makes the green LEDs have a high current efficiency of 66.1 cd A^−1^.

In this review, the structural types of 2D perovskite are reviewed. Then, the unique optical properties of 2D perovskite are discussed as the advantages of the emitting layer of LEDs. In addition, the effects of different types of organic cations on devices and the design principles of high-efficiency 2D PeLEDs are summarized. Finally, we emphasize that 2D PeLEDs have great potential in the fields of display and solid-state lightings.

## Characteristics of 2D Perovskite Materials

### Structure of 2D Perovskite Materials

**Fig. 1:**
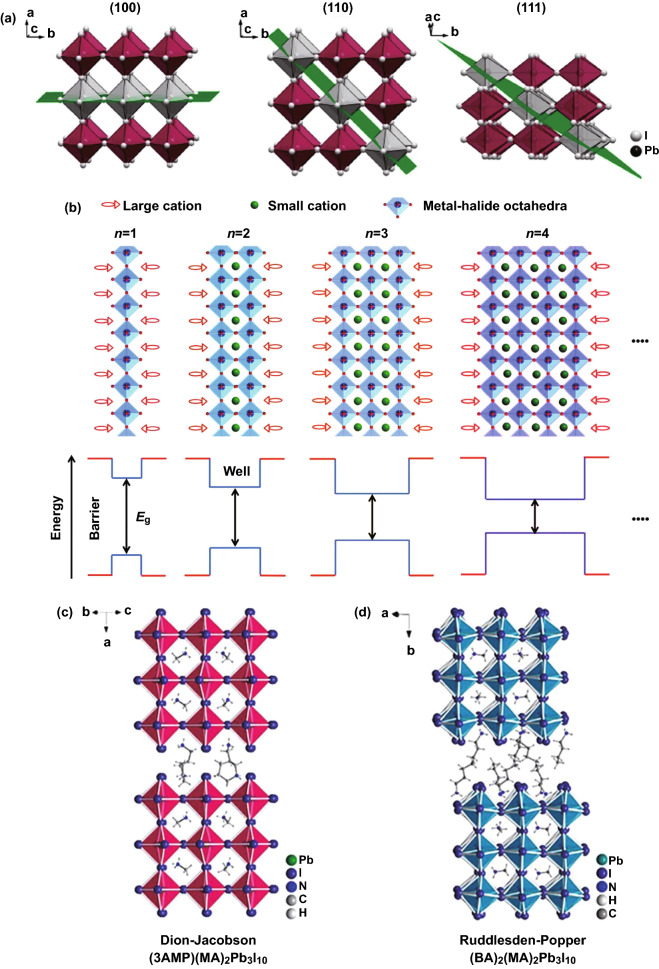
**a** Schematic diagram of 2D perovskite series with different orientations. Copyright 2018 American Chemical Society [47]. **b** Schematic diagram of quantum wells perovskites. Copyright 2020 Wiley-Blackwell [65]. Comparison between **c** Dion− Jacobson phase and **d** Ruddlesden–Popper phase for halide perovskite. 3AMP = 3-(aminomethyl)piperidinium, 4AMP = 4-(aminomethyl)piperidinium, MA = methylammonium, BA = butylammonium. Copyright 2018 American Chemical Society [50]

2D perovskite materials with (100)-oriented, (110)-oriented and (111)-oriented structures are formed by cutting along the specific (hkl) planes of the corresponding 3D perovskite structures (Fig. 1a) [46–48]. The most studied are the (100)-oriented 2D perovskites with K_2_NiF_4_ or RbAlF_4_ crystal structure, and the general formula is A′_2_A_n-1_B_n_X_3n+1_. The general formula of (110)-oriented 2D perovskite is A′_2_A_m_B_m_X_3m+2_. Because octahedrons are usually highly distorted, there will be many interesting physical phenomena at room temperature, such as self-trapping exciton and white light emission [49]. The general formula of (111)-oriented 2D perovskite is A′_2_A_q-1_B_q_X_3q+3_(q > 1).

At present, (100)-oriented perovskites are the most widely studied 2D perovskites, which can be further divided into Ruddlesden–Popper (RP) and Dion–Jacobson (DJ) phases. The general formulas of RP and DJ perovskites are A′_2_A_n−1_B_n_X_3n+1_ and A′′A_n−1_B_n_X_3n+1_, respectively, where A’ represents a large aromatic or aliphatic alkyl ammonium cation (monovalent) and A′′ is divalent cation. A is a small cation like CH_3_NH_3_^+^, Cs^+^, CH(NH_2_)_2_^+^, B refers to a divalent metal cation (Pb^2+^, Sn^2+^, Cu^2+^, Cd^2+^, Zn^2+^), and X represents an anion, such as Cl^−^, Br^−^, I^−^, SCN^−^ and F^−^. For the DJ phase (3AMP)(MA)_2_Pb_3_I_10_, the layers overlap completely without any displacement (Fig. 1c) [50]. Different from DJ phase, the layers of (BA)_2_(MA)_2_Pb_3_I_10_ show (1/2, 1/2) displacement in RP phase (Fig. 1d) [51]. In addition, the interlayer distance between DJ and RP phase is quite different. Generally, the interlayer distance of RP phase is larger than that of DJ phase. RP phase has double-layer organic cations, which increases the interlayer distance.

The (110)-oriented structures are the most distorted and uncommon, and few cations can stabilize them. Generally, these cations have small ionic radii and highly symmetrical structures [52]. The (100)-oriented 2D perovskites consist of flat perovskite sheets, while the (110)-oriented perovskites have corrugated layers. The structures can be defined as 2 × 2, 3 × 3, 4 × 4, etc., according to the corrugation length. These perovskites with roof shape are named “*n* × *n*,” where *n* is the number of octahedrons that make up half of the roof.

Different from other layered perovskites, (111)-oriented perovskites are actually a kind of defective perovskites. The formation of layered perovskites is due to the introduction of vacancies rather than large organic cations. The (111)-oriented perovskites belong to M-site-deficient, and the general formula is A’_2_A_q-1_B_q_X_3q+3_ (*q* > 1). The (111)-oriented perovskites can be obtained by cutting along the volume diagonal of 3D perovskite cell, which selectively “eliminates” the metal sites in the cutting process. Thus, if the B site contains only divalent metal ions, there is no way to form (111)-oriented perovskites. When B site is the same metal ion (*q* = 2), some common (111)-oriented perovskite materials such as FA_3_Bi_2_Br_9_ [53], Cs_3_Bi_2_Br_9_ [54], Cs_3_Sb_2_I_9_ [55], Rb_3_Sb_2_I_9_ [56] and MA_3_Bi_2_I_9_ [57].

### *n* Values in the 2D Perovskites

**Fig. 2:**
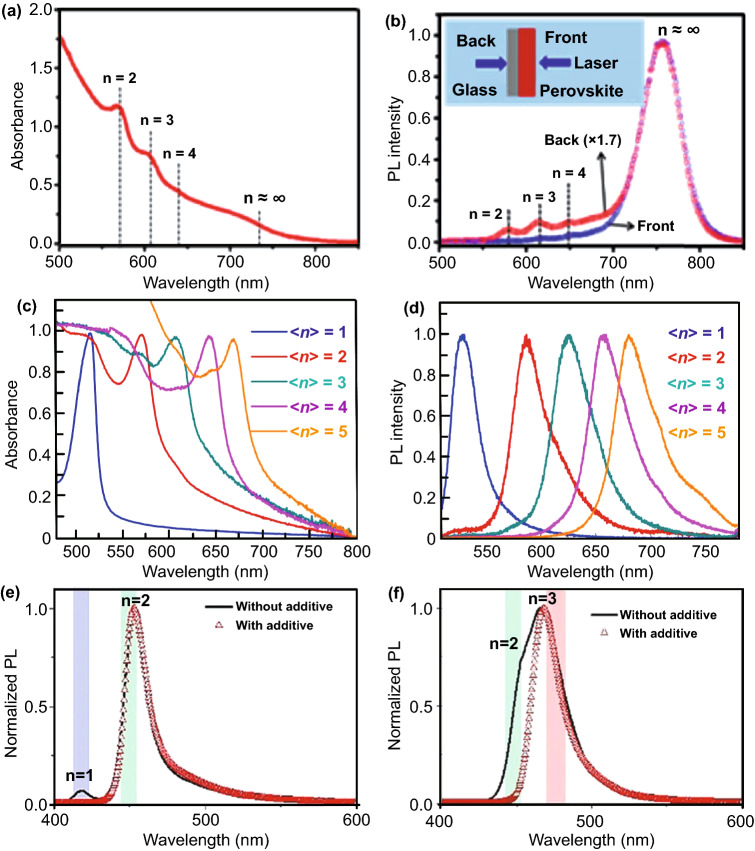
**a** UV–Vis absorption spectra of (BA)_2_(MA)_3_Pb_4_I_13_ perovskite film. **b** Comparison of the emission spectra of the 2D perovskite film illuminated from the front and back sides of the film. Copyright 2018 American Chemical Society [59]. **c** Normalized UV–Visible absorption spectrum of the phase-pure films. **d** Normalized PL spectrum of the phase-pure films. Copyright 2020 Springer Nature [60]. Photoluminescence spectra (375 nm excitation) for the system using **e** BABr: MABr: PbBr_2_ = 1.5: 0.5: 1 and **f** BABr: MABr: PbBr_2_ = 1.1: 0.5: 1 molar ratio precursor. Copyright 2021 Wiley–VCH GmbH [62]

The value of *n* in the formula is defined as the number of [BX_6_]^4−^ octahedral layers, which can be achieved by adjusting the stoichiometry of the precursor solution. The band gap, carrier mobility and *E*_*b*_ of 2D perovskite are all related to *n* value. At present, in most reports, researchers have assumed that there is a specific *n* value in 2D perovskite thin films. However, the 2D perovskites prepared according to the formula are usually mixtures with different *n* values, even though the purpose is to prepare pure 2D perovskites [58]. Jin et al. prepared 2D perovskite (BA)_2_(MA)_3_Pb_4_I_13_ with nominal “*n* = 4,” but found that the sample is a mixture of multiple perovskite phases. As shown in Fig. 2a, the absorption peaks at ∼572, 608, 645 and 750 nm can be assigned to pure phase (BA)_2_(MA)_*n*−1_Pb_n_I_3n+1_ with *n* = 2, 3, 4 and ∞, respectively [59]. Combined with the absorption and PL spectra (Fig. 2a, b), it can be inferred that the bottom is conducive to the formation of low *n* phase, and high-*n* phase is easy to form at the top. For display and lasing applications with high color purity, it is necessary to accurately control 2D perovskites with specific *n* value, which can be obtained by growing single crystal. However, due to the fast nucleation rate of perovskites, the precise preparation of perovskite films with specific *n* value is still a worldwide scientific problem. Recently, Huang et al. introduced *n*-butylamine acetate to replace the traditional *n*-butylamine iodide to obtain phase-pure thin films. Figure 2c, d shows the absorption and PL spectra of the phase-pure RP perovskite films, which have obvious single peaks compared with the previous studies. The peak positions of the films are red shifted with the increase of *n* value [60, 61]. Chiu et al. [62] used tin-based and lead-based perovskites as research objects, added carboxylic acid in the anti-solvent, through the kinetic control of 2D perovskites nucleation process, inhibited the formation of thermodynamic favorable small *n* value and improved the selectivity for 2D perovskites with specific *n* value. It can be seen from Fig. 2e that the addition of hexanoic acid significantly suppress the formation of (BA)_2_PbBr_4_ (*n* = 1). Interestingly, the control of hexanoic acid is not limited to the synthesis of *n* = 2 phase-pure perovskites. The *n* = 3 perovskite nanoplates can also be obtained by reducing the molar ratio of butylamine hydrobromide (Fig. 2f).

Compared with 3D perovskites, 2D perovskites not only have dimensional changes, but also have natural quantum well structures [63]. As shown in Fig. 1b, n represents the number of [BX_4_]^2−^ octahedron layers, which can determine the well width and band gap. The larger the *n* value, the smaller the band gap. The [BX_4_]^2−^ sheet can be regarded as a potential well, and the large organic cation acts as a potential barrier. And the barrier width is determined by the radius of the large organic cation. Through the self-assembly from bottom to top, the organic–inorganic layers are arranged alternately to form multiple quantum well structures naturally. In addition, the *E*_*b*_ of 2D perovskites are larger than that of 3D perovskites due to the large difference of dielectric constants between organic and inorganic layers. Generally, the 2D layered perovskites contain many different components (marked by *n* value). The quantum wells have an ultra-fast energy transfer process; the transfer efficiency is close to 100% (sub nanoscale), which effectively inhibits exciton quenching and makes the quantum well films exhibit high photoluminescence quantum efficiency (PLQY) under low excitation [64, 65]. Di et al. [44] achieved PeLED comparable to organic light-emitting diodes (OLEDs) and quantum dot light-emitting diodes (QD LEDs) by enhancing outcoupling with polymer additives, with EQE as high as 20.1% at 0.1–1 mA cm^−2^.

### Charge-Carrier Recombination Kinetics

**Fig. 3:**
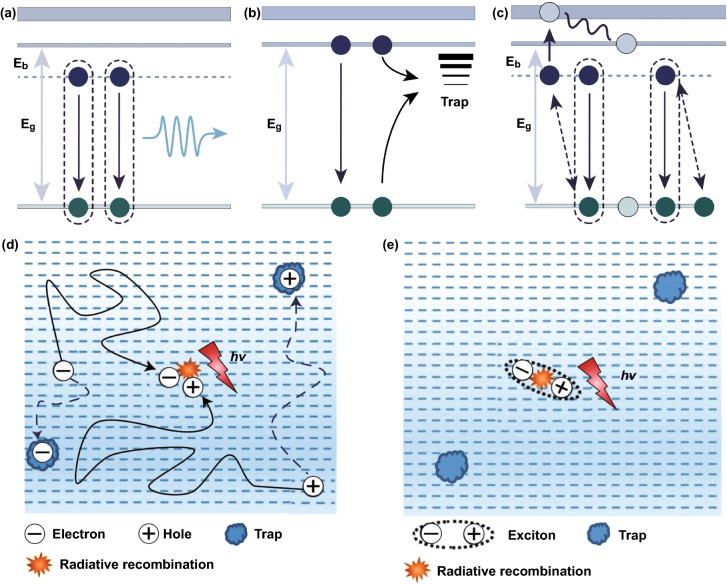
**a** Exciton recombination. **b** Trap-assistant recombination. **c** Auger recombination. Copyright 2021 Springer Nature [72]. A schematic illustration of charge-carrier recombination **d** in 3D perovskites and **e** in 2D perovskites. Copyright 2020 Springer Nature [71]

From the material point of view, the PLQY and photoexcited charge-carrier lifetime of perovskite materials are closely associated with the realization of efficient PeLED [30, 66, 67]. In Eq. 1, *R*_*r*_ and *R*_*nr*_ are radiative and non-radiative recombination rates, respectively. In perovskite, radiative recombination can be band-to-band recombination or exciton recombination, while non-radiative processes are Shockley–Read–Hall (SRH) recombination and multiparticle interaction recombination (three body Auger recombination) (Fig. 3). The kinetics of carrier recombination in 3D perovskites can be summarized by Eq. 2. *n* is the charge-carrier density, *t* is the time, *k*_1_ is the trap-assisted monomolecular recombination rate constant (SRH), *k*_2_ is the bimolecular recombination rate constant (band-to-band recombination), and *k*_3_ is the three body Auger recombination rate constant; among them, *k*_1_ is usually affected by carrier confinement and defects, and *k*_2_ and *k*_3_ are intrinsic values. Carrier density determines which recombination is dominant. It is well known that free carriers are dominant in 3D perovskites because of their small *E*_*b*_, which is close to room temperature thermal energy. This indicates that the excitons will dissociate into free carriers at room temperature, so the radiative recombination in 3D perovskite is mainly controlled by the band-to-band recombination k_2_ [68]. As expressed in Eq. 2, the relationship between carrier recombination kinetics and carrier density is inseparable in 3D perovskites. Due to the slow band-to-band recombination rate (Fig. 3d), trap-assisted monomolecular recombination (*k*_1_) is dominant at low carrier density, and free carriers are easy to be trapped by defects. If the carrier density is high, the traps are full of free carriers and reach saturation states, which promotes bimolecular radiative recombination and improves PLQY.

1 $${\text{PLQY}} = \frac{{\sum R_{r} }}{{\sum R_{r} + \sum R_{nr} }}$$

2 $$- \frac{{{\text{d}}n}}{{{\text{d}}t}} = k_{1} n + k_{2} n^{2} + k_{3} n^{3}$$

It is a pity that the charge-carrier density in typical 3D PeLED is usually less than 10^15^ cm^−3^, which severely limits the rate of radiative recombination [48]. Reducing the size or dimension (such as 2D, 1D) of perovskite crystals can effectively increase the charge-carrier confinement [69, 70]. From the above point of view, 2D perovskites are an ideal candidate for PeLED. Unlike 3D perovskites, 2D perovskites have exciton binding energies as high as several hundred milli-electron volts. As shown in Fig. 3e, the excitons are stable and difficult to separate into free carriers at room temperature, which directly leads to the rapid radiative monomolecular recombination rates [41], and hence, the relationship between the carrier recombination dynamics and the carrier density changes correspondingly. Equation 2 is not suitable for 2D perovskites, and this relationship is shown in equation [71]. Where *k*_trap_ is non-radiative monomolecular recombination rate constant, *k* is radiative monomolecular recombination rate constant and *k*_2_ is the rate constant of Auger recombination. From Eq. 3, it can be seen that the influence of carrier density on carrier recombination kinetics is reduced. In 2D perovskite, excitonic recombination not only competes with non-radiative monomolecular recombination, but also competes with Auger recombination.

3 $$- \frac{{{\text{d}}n}}{{{\text{d}}t}} = (k_{{{\text{trap}}}} + k)n + k_{2} n^{2}$$

### Advantages of 2D Perovskite as Light-Emitting Layer

It is well known that high PLQY is the key factor to realize efficient PeLEDs. The *E*_*b*_ of 2D perovskites is large, which promotes the enhancement of radiative recombination rate. PLQY is also strongly dependent on defect density. The decrease of defect density can reduce the possibility of non-radiative recombination. In essence, the problem of low luminescence efficiency of materials is solved. Furthermore, the high-quality film morphology and balanced bipolar charge injection are the key factors to determine the EQE of PeLEDs, because the compact films can effectively reduce the leakage current. It was found that large organic cations are favorable for the formation of uniform pinhole-free films in 2D perovskite [73].

#### Excitonic Characteristics and Ultra-Fast Energy Transfer

**Fig. 4:**
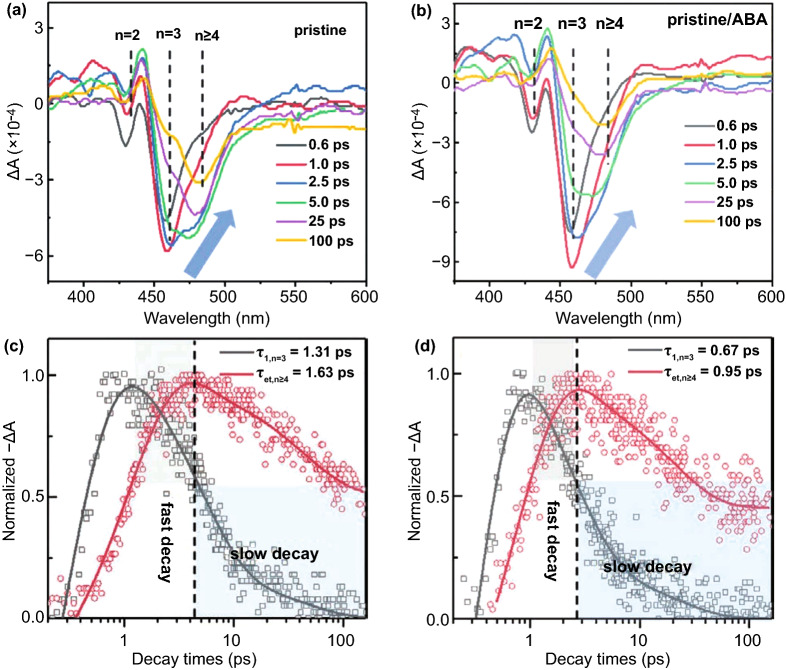
TA spectroscopy of **a** the pristine and **b** pristine/ABA perovskite films. TA kinetics probed at selected wavelengths (453 and 481 nm) for **c** pristine and **d** pristine/ABA perovskite films. Copyright 2020 Wiley-Blackwell [77]

3D perovskites usually have a small *E*_*b*_, which is unfavorable for LEDs [74]. Compared with 3D perovskites, 2D perovskites are affected by quantum confinement and dielectric confinement [75]. The effect of quantum confinement origin the close thickness of the [BX_6_]^4−^ octahedral layers to the Bohr radius. The dielectric constant offset between organic cations and [BX_6_]^4−^ octahedral layers results in the dielectric confinement effect. These effects are beneficial to the formation of bound excitons. Besides, the natural quantum well structure has ultra-fast energy transfer, so the carrier density increases and the shallow defects which affect the luminescence efficiency are filled. In 2D perovskites, the trap-assisted monomolecular recombination lost its competitiveness, and the first-order excitonic radiative recombination is dominant, and PLQY is improved [31]. Similar to the host–guest systems of OLEDs, energy transfer can occur among different samples with *n* values. The cascade energy transfer from the wide bandgap (small *n* value) to the narrow bandgap (large *n* value) can effectively avoid the concentration quenching and dramatically improve the radiative recombination rate in the maximum *n* value [42, 76]. Unfortunately, the quasi-2D perovskites are a mixture of many phases (especially a large number of small *n* phases), resulting in inefficient energy transfer. Choy et al. introduced a bifunctional organic ligand (amino and carboxyl group) to weaken the van der Waals gap, which can contribute to the efficient energy transfer of perovskite films. As shown in the ultra-fast transient spectroscopy (TA) spectra (Fig. 4a, b), three distinctive ground-state bleach peaks corresponding to *n* = 2, 3 and ≥4 are observed at 428, 453 and 481 nm, respectively. With increasing decay time, the ground-state bleach peak value of the small *n* phase gradually decreases, and that of the large *n* phase (*n* ≥ 4) gradually increases, indicating that carriers transfer from small *n* phase to large *n* phase. Further, *n* = 3 phase and *n* ≥ 4 phase are selected as the research objects, and the kinetic multi-exponential function fitting is carried out. Both *n* = 3 (*τ*_1_ = 0.67 ps) and *n* ≥ 4 (*τ*_et_ = 0.95 ps) in the pristine/ABA perovskites show smaller values, which are about half of the pristine perovskites (*τ*_1, *n* = 3_ = 1.31 ps; *τ*_et, *n* ≥ 4_ = 1.63 ps), implying that more efficient energy transfer is achieved (Fig. 4c, d). Moreover, the carrier transfer is completed in picoseconds [77]. Lee et al. [43] also observed a similar result, and the enhancement of energy transfer is favorable to reduce the probability of non-radiative recombination, thus improving the device performance. Although the mechanism of photogenerated electron and hole transfer is still controversial, the rapid carrier transfer process can compete with trap-assisted monomolecular non-radiative recombination, which makes the quasi-2D perovskite structure beneficial to enhance PLQY [78, 79].

#### Dense Film Morphology

**Fig. 5:**
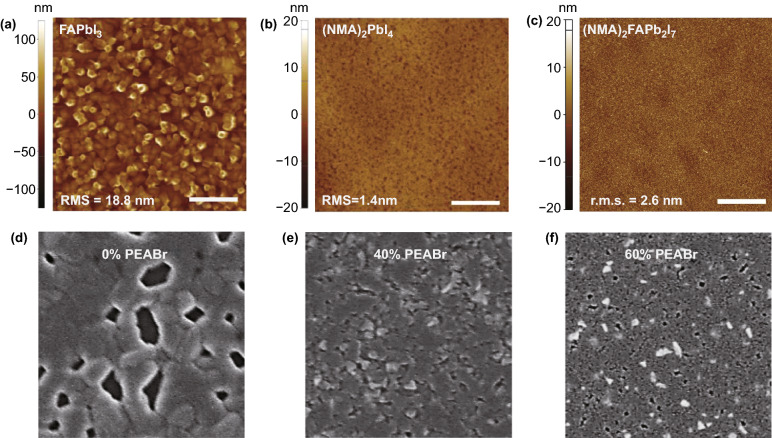
AFM height images of **a** FAPbI_3_ **b** (NMA)_2_PbI_4_ and **c** (NMA)_2_FAPb_2_I_7_ films (scale bar 5 μm). Copyright 2016 Springer Nature [42]. SEM images of perovskite films with d 0% PEABr e 40% PEABr and f 60% PEABr. Copyright 2019 Springer Nature [84]

The morphology of perovskite films is a key factor to determine the performance of PeLEDs. Generally, 3D perovskite thin films have rough surface and are prone to a large number of pinholes, leading to the increase of leakage current and obvious thermal effect, which will be resulting in the decrease of device efficiency and the shortening of device life [80, 81]. The results show that 2D perovskites have better film morphology than 3D perovskites. Organic cations can improve the morphology of 3D perovskite films and promote the formation of dense pinhole-free films [78, 82, 83]. For example, the root-mean-square roughness of 3D FAPbI_3_ perovskite film is 18.8 nm. Surprisingly, the root-mean-square roughness of 2D (NMA)_2_PbI_4_ (*n* = 1) and (NMA)_2_FAPb_2_I_7_ (*n* = 2) films is 1.4 and 2.6 nm, respectively (Fig. 5a–c) [42]. In addition, Cao et al. improved the surface coverage of CsPbCl_0.9_Br_2.1_ film by introducing PEABr, with only a few small pinholes (Fig. 5d–f). More importantly, if PEABr is replaced by other amines, a similar phenomenon will be observed [84].

#### Lower Efficiency Roll-Off

**Fig. 6:**
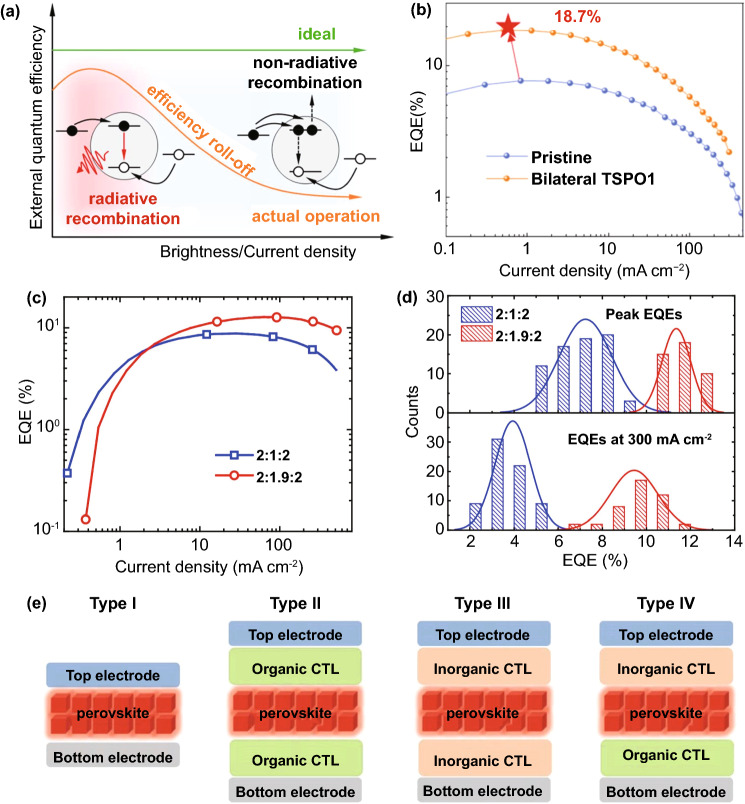
**a** Efficiency roll-off under ideal and actual PeLEDs operating conditions. Copyright 2020 American Chemical Society [100]. **b** EQE of original and bilateral-passivated device. Copyright 2020 Springer Nature [87]. **c** EQE versus current density. **d** Histograms of peak EQEs and EQEs at 300 mA cm^−2^. Copyright 2018 Springer Nature [91]. **e** Schematic diagrams of the four device structures of PeLEDs. Copyright 2019 Wiley–VCH Verlag Copyright 2019 Wiley–VCH Verlag [85]

At present, the EQE of classical perovskite material CsPbX_3_ (*X* = Cl^−^, Br^−^, I^−^) has exceeded 20% [85]. However, as shown in Fig. 6a, with increasing brightness and current density, the EQE of devices tends to decrease, which is called efficiency roll-off. In particular, due to the exciton effect, the efficiency rolls off of perovskite quantum dots are very serious at high current density [86]. Zeng et al. [87] used organic molecules to passivate the top and bottom of perovskite quantum dot film, which improved the stability of PeLEDs and effectively suppressed the non-radiative recombination. The EQE was as high as 18.7% at low current density. Unfortunately, at a high current density of 100 mA cm^−2^, the EQE value is only 32% of the maximum value (Fig. 6b). Sargent et al. [88] proposed to construct a bipolar shell composed of inner anion shell on the surface of perovskite quantum dots. More than 90% of PLQY blue quantum dot films were obtained. Surprisingly, the maximum EQE value is 12.3%, but there is also a large efficiency roll-off value. For most devices, the decrease of EQE at high current density is due to the combination of charge injection imbalance, Auger-induced luminescence quenching and Joule heating [89, 90]. Perovskite quantum dots LEDs have severe efficiency roll-off because of Auger recombination at low current density. However, in quasi-2D perovskite, Auger recombination can be suppressed by reducing the local carrier density in the quantum well. Huang et al. [91] changed the width of perovskite quantum wells by controlling the proportion of organic cations without reducing the luminescence performance. The results show that increasing the well width can significantly suppress the luminescence quenching effect. As shown in Fig. 6c, the maximum EQE values of 2:1:2 and 2:1.9:2 devices are 8.7% and 12.7%, respectively. Furthermore, the efficiency roll-off of 2:1.9:2 device is effectively suppressed; even at 500 mA cm^−2^, it can still maintain 10% of the peak value. In addition, compared with 2:1.9:2 device (5.7%), the relative standard deviation of 2:1:2 device is larger (16.3%). Under the same current density (300 mA cm^−2^), the average EQE value of 2:1.9:2 device is more than twice that of 2:1:2 device (Fig. 6d). Although adjusting the well width can effectively reduce the efficiency roll-off, compared with the 3D PeLEDs, quasi-2D PeLEDs also suffer from serious efficiency roll-off. The strategies of reducing the roll-off effect of quasi-2D PeLEDs will be discussed in details in the next section.

## Strategies for Boosting the Performance of PeLEDs

### Suppression of Efficiency Roll-Off

As we all know, quasi-2D perovskite materials are expected to be candidates for the next-generation display applications [58, 92]. Unfortunately, the quasi-2D perovskite materials suffer from serious efficiency roll-off, which is mainly caused by the charge injection imbalance, Auger recombination and Joule heating [93].

#### Balance of Charge Injection

High PLQY is the key factor to realize efficient and stable 2D PeLEDs. Other key elements include effective charge injection, defect passivation and ion migration suppression. Currently, for PeLEDs devices, there is no unified name for the device structures, which are usually divided into formal and inverted device structures. Rogach et al. adjusted the classification of PeLEDs device structures on the basis of Cd-based QD LEDs. As shown in Fig. 6e, type I has no charge transport layer, charge transport layers of type II are all organic polymers/small molecules, charge transport layers of type III are all inorganic, and organic and inorganic molecules of type IV are used as charge transport layers [85]. For 2D PeLEDs, the device structures of type I and III are rarely reported, while type II and IV are mostly studied.

**Fig. 7:**
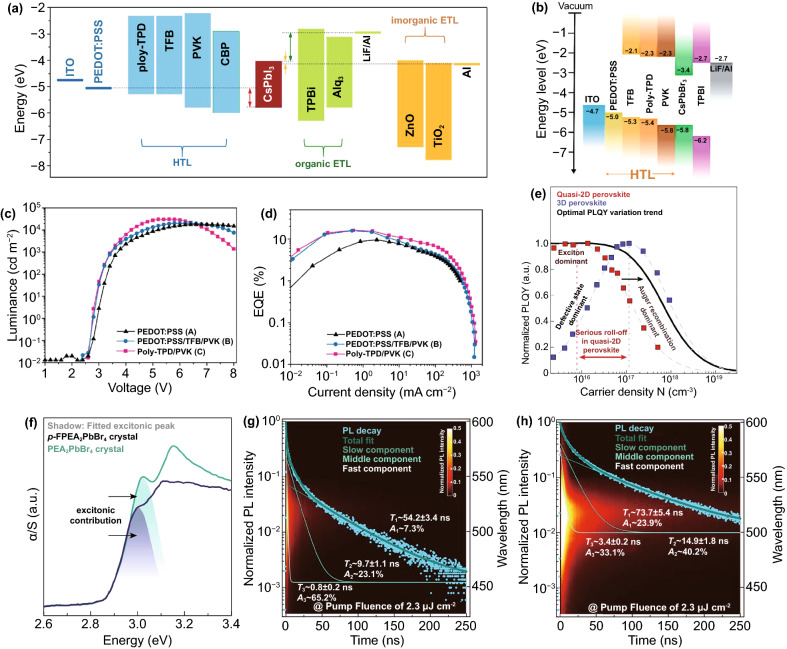
**a** Schematic diagram showing energy levels of typical electrodes, charge transport materials and CsPbI_3_. Copyright 2020 American Chemical Society [100]. **b** Energy level diagrams of quasi-2D PeLEDs and different HTLs. **c** L-V and **d** EQE-J of PEDOT: PSS, PEDOT: PSS/TFB/PVK, poly-TPD/PVK. Copyright 2020 American Chemical Society [102]. **e** Experimental PLQYs of quasi-2D perovskite and 3D perovskite films as a function of carrier density. **f** Absorption spectra of PEA2PbBr4 and p-FPEA_2_PbBr_4_ single crystals. Time- and wavelength-dependent photoluminescence mapping under a pump fluence of 2.3 μJ cm^−2^ for **g** PEA and **h** p-FPEA films. Copyright 2021 Springer Nature [72]

The charge injection imbalance is related to the injection barrier [94], carrier mobilities [95] and defect density [96]. The relationship between barrier height and electric current can be seen from Eqs. 4 and 5

4 $$J \propto E^{2} \exp \left( {\frac{ - b}{E}} \right)$$

5 $$b = \frac{{8\pi \sqrt {2m^{*} \varphi^{\frac{3}{2}} } }}{3qh}$$

where *J* is the current density, *E* is the electric field, *φ* is the barrier height, m* is the carrier effective mass, *q* is the element charge, and *h* is the Planck constant [97]. The work function of the cathode is different from that of the conduction band bottom of the electron transport layer (ETL). Similarly, the difference also exists in the work function of the anode and the top of the valence band of the hole transport layer (HTL), resulting in potential barrier. To overcome the barrier, the charge carrier must have enough energy. The relationship between barrier height and luminous efficiency can be seen in Eq. 6.

6 $${\text{Efficiency}} \propto \exp \frac{{ - r\varphi^{\frac{3}{2}} }}{v}$$

where *V* is applied bias and *γ* is constant. The turn-on voltage and luminous efficiency of the device are affected by the barrier heights of the interfaces and the semiconductor energy levels of each layer. The turn-on voltage is closely associated with the flat band conditions determined by the work functions of cathode and anode. For 2D PeLEDs, it can be seen from Eq. 6 that the charge injection with a small energy barrier is a necessary condition to obtain low operating voltage and high luminous efficiency. Also, the stability of 2D PeLED is also going hand in hand with effective carrier injection. Invalid carrier injection will lead to charge imbalance and space charge accumulation in perovskite emitting layer. Thus, it is very important to match the energy levels at the interfaces [98]. For most PeLEDs, high electron injection efficiency and low hole injection efficiency lead to the imbalance of charge injection, which seriously affects the performance of PeLEDs [99, 100]. As shown in Fig. 7a, taking CsPbI_3_ as an example, there is a high barrier between HTL and emitting layer, and holes need to overcome it to successfully transport to emitting layer (red arrow). However, ETL is different from HTL. For inorganic ETL, only a very small barrier needs to be overcome to reach the perovskite layer (yellow arrow). Furthermore, for organic ETL, electrons can be easily transported to the emitting layer due to the additional driving force (green arrow) [100]. At a low current density, more radiative recombination can be maintained with less carrier injection. At a high current density, the number of carriers injected increases, and the electron hole pairs are easily quenched between the HTL and the emitting layer due to the mismatch of energy levels [101]. Lin et al. established “energy ladder” in HTL to solve the problem of charge injection imbalance (Fig. 7b). It can be seen from Fig. 7c that the maximum luminance of multilayer HTL devices (PEDOT: PSS/TFB/PVK, poly-TPD/PVK) is 20342 and 31,012 cd m^−2^, respectively, while the maximum luminance of single-layer HTL device (PEDOT: PSS) is only 18,154 cd m^−2^. The establishment of “energy ladder” is helpful to improve the efficiency of hole injection and reduce the turn-on voltage. It is gratifying that the EQEs of multilayer HTL devices have also been greatly improved (Fig. 7d), and the efficiency roll-off of poly-TPD/PVK is suppressed at the current density of 0.01–1000 mA cm^−2^ [102].

Although balanced charge injection can suppress the efficiency roll-off, there is still a serious efficiency roll-off at a high current density, which may be due to the existence of long organic chains, resulting in poor charge transport, or the increase of Auger recombination rate due to the quantum well structures.

#### Reducing of Auger Recombination

Auger recombination is affected by carrier density, as shown in Eq. 7, where *g*_eeh_ is the Coulomb enhancement factor, and the value is associated with E_b_. $k_{{{\text{Auger}}}}^{0}$ is the band-to-band Auger recombination rate (non-interact particles), C_*n*_ is the Auger coefficient, and *n* and *P* are the electron density and hole density, respectively.

7 $$k_{{{\text{Auger}}}} = g_{{{\text{eeh}}}} k_{{{\text{Auger}}}}^{0} = g_{{{\text{eeh}}}} C_{n} n^{2} p$$

As shown in Fig. 7e, the black curve is an ideal evolution trend of PLQY. The curve trend of the quasi-2D perovskites is highly similar to that of the black curve. Even at a low current density, the quasi-2D perovskites can obtain high PLQY. However, compared with 3D perovskites, Auger recombination of quasi-2D perovskites is very severe. That is because the carrier density of 3D perovskites is lower than that of quasi-2D perovskites [41, 103]. From Eq. 7, it can be concluded that the Auger recombination rate is positively related to the third power of carrier density, so the Auger recombination rate of quasi-2D perovskites increases. The other reason is due to the enhanced electron hole interaction, which leads to the non-uniform distribution of carriers [104, 105]. Yuan et al. increased the dielectric constant of organic cations to weaken the “dielectric confinement effect,” which not only significantly reduced *E*_*b*_, but also did not change the energy transfer efficiency. Using highly polarized *p*-FPEA^+^ to replace the A site in the classical quasi-2D perovskite material PEA_2_MA_n−1_Pb_n_Br_3n+1_, the molecular dipole moment is increased (simulated by density function theory), which is conducive to charge separation. Strong evidence for the decrease of *E*_*b*_ is found in the optical absorption spectra. As shown in Fig. 7f, PEA_2_PbBr_4_ has an obvious excitonic peak at about 3.08 eV, while only a small kink (3.04 eV) is observed in *p*-FPEA_2_PbBr_4_. Moreover, the *E*_*b*_ values are estimated quantitatively by temperature-dependent photoluminescence measurements. And the *E*_*b*_ values of PEA_2_PbBr_4_ and *p*-FPEA_2_PbBr_4_ are 347 and 195 meV, respectively. To further strengthen this conclusion, multi-exponential fitting is used to analyze the carrier dynamics at a high current density (up to ~1 × 10^17^ cm^−3^). The fastest decay is associated with Auger recombination. The fast decay times of Auger recombination for PEA and *p*-FPE samples are 0.8 and 3.4 ns, respectively (Fig. 7g, h). Thus, Auger recombination rate decreased significantly. Unfortunately, while Auger recombination decreased, PLQY also decreased significantly. On the basis of previous study, molecular passivation (another strategy) was used to reduce trap-assistant recombination. Finally, the device showed a record luminance (82,480 cd m^−2^) [72].

### Passivation of Surface Defects

**Fig. 8:**
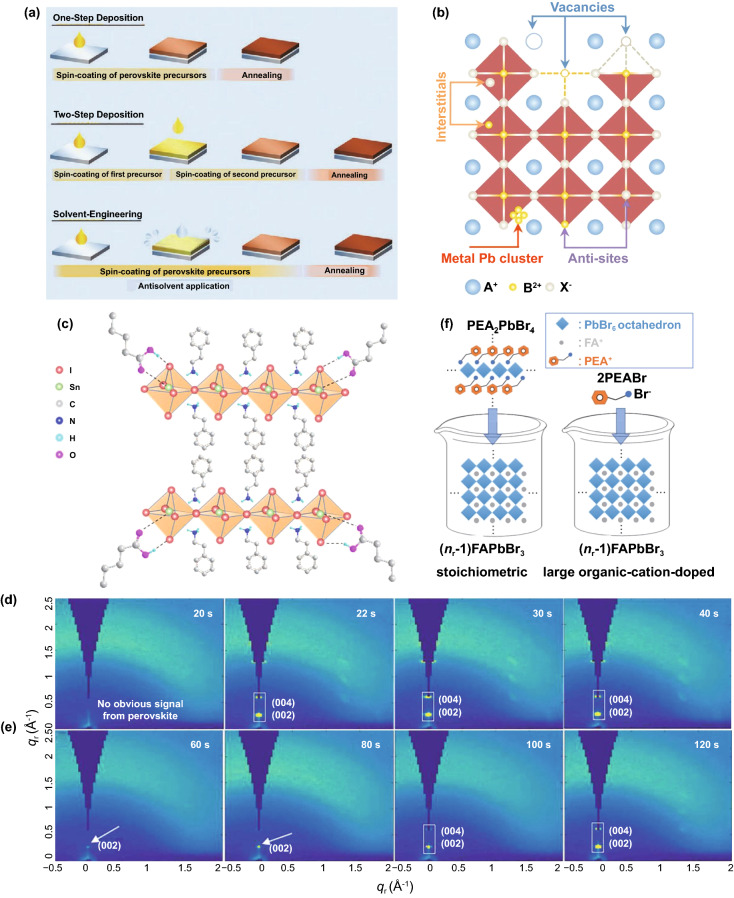
**a** Schematic diagram of perovskites deposition by solution processing. Copyright 2020 Wiley–VCH Verlag [107]. **b** Defects of perovskites include vacancies, anti-sites, interstitials and metal clusters. Copyright 2020 Springer Nature [71]. **c** Diagram of hydrogen bonding and coordination bonding interactions between VA and PEA_2_SnI_4_ crystals. In situ GIWAXS measurements of spin-coated PEA2SnI4 **d** without and **e** with VA at different times. Copyright 2020 American Association for the Advancement of Science [114]. **f** Illustration of perovskite precursor solutions prepared by controlling stoichiometry and adding organic ammonium salts. Copyright 2020 Wiley–VCH Verlag [115]

At present, a large number of research reports regard solution processing as the deposition method of PeLEDs [106, 107]. Solution processing has significant advantages, such as high cost-effectiveness, simple process and no need for a complex vacuum system [108, 109]. It has broad application prospects in PeLEDs. General solution-processing methods include one-step, two-step and solvent engineering methods (Fig. 8a) [107]. Solvent engineering is the most widely used method so far, which is based on the one-step method, adding anti-solvent to promote the crystallization of perovskite films [110]. However, solution-processing perovskite materials suffer from severe non-radiative recombination (caused by traps), which seriously limits the application and development of PeLEDs [111, 112]. Trap states are generally considered to be related to ionic defects, such as vacancies, anti-site occupations and interstitials (Fig. 8b) [71]. Using passivation agents to passivate defects is a very effective method, which has been determined to reduce the defect density and non-radiative recombination of perovskites. Usually, the passivation agents are added at the beginning of the reaction or in the post-treatment. Common passivation agents include Lewis acid/base and organic halide salts [113]. For example, Sargent et al. produced high-efficiency, lead-free PeLED by adding valeric acid (VA). The EQE is 5%, and the half-life is exceeding 15 h. That is so due to strong forces, of hydrogen bonds (O=C–OH…I^−^) and coordination bond (C=O…Sn^2+^) between VA and PEA_2_SnI_4_ thin films (Fig. 8c). The existence of the interaction forces inhibits the rapid nucleation and slows down the crystallization rate, thus forming a uniform pinhole-free morphology (Fig. 8d, e). And VA can also be used to inhibit the oxidation of tin in the process of film formation [114]. Moreover, Adachi et al. systematically compared the effects of controlling stoichiometry and adding organic ammonium salts on the surface defects and domain distribution of the films with quasi-2D perovskites (Fig. 8f). The results show that PEA^+^ cations in the stoichiometric control method mainly play the role of passivation. In contrast, adding a large amount of PEABr in 3D perovskite precursor can not only passivate the surface defects but also inhibit the appearance of small *n* values, which is helpful to the formation of quasi-2D perovskite domains. It is well known that low-order domains damage the performance of PeLEDs. Therefore, perovskite films with organic ammonium salts have higher defect passivation efficiency and higher EQE [115].

### Critical Role of Organic Cations for PeLEDs

#### Reasonable Selection of Organic Cations

It is universally acknowledged that 2D perovskites can still show better stability than 3D perovskites when exposed to moisture, which is due to the hydrophobicity of organic cations. And there is a strong interaction between the organic cations and the capped organic molecules. Moreover, the chain lengths and π − π stacking of organic cations affect the *n* distribution of 2D perovskites [116–118]. A variety of organic cations, such as butylammonium (BA) [119], phenylethylammonium (PEA) [120], 1-naphthylmethylammonium (NMA) [120] and benzimidazolium (BIZ) [121], have been studied (Table 1). Unfortunately, most of the research reports focus on a specific ion. There are some questions about which organic cation should be selected, and the device design is not reasonable.

**Table 1:** Various large organic cations in PeLEDs

Cation	Emitter material	EL (nm)	EQE_max_ (%)	L_max_ (cd m^−2^)	Year	Refs
Propylammonium (PA^+^)	PA_2_CsPb_2_I_7_: CsPb(Br,Cl)_3_	693	/	/	2018	[127]
	PA_2_(CsPbBr_3_)_*n*−1_PbBr_4_	492	1.45	5737	2019	[128]
	PA_2_(CsPbBr_3_)_*n*-1_PbBr_4_	~ 505	3.6	7320	2018	[129]
Butylammonium (BA^+^)	BA_2_(CsPbBr_3_)_*n*−1_PbBr_4_-PEO	514	8.42	33,533	2018	[130]
	BA_2_Cs_n−1_Pb_n_ (Br/Y)_3n+1_	506	10.1	3810	2018	[131]
	BA_2_FA_2_Pb_3_Br_10_ (*n* = 3)	543	14.6	24,100	2018	[132]
	*n*-BABr-MAPbBr_3_	/	17.5	48,668	2019	[133]
	BA_2_Cs_n–1_Pb_n_Br_3n+1_	512	20.5	13,400	2021	[134]
	(BA)_2_Cs_n-1_Pb_n_Br_3n+1_	/	8.44	3610	2020	[119]
	BA_2_Cs_4_Pb_5_Br_16_	512	16.35	6785	2020	[135]
	BA_2_MA_n−1_Pb_n_Br_3n+1_	526.9	/	~ 103	2020	[123]
Octylamine	(C_8_H_17_NH_3_)_2_(CH(NH_2_)_2_)_m−1_Pb_m_Br_3m+1_	540	5	/	2018	[136]
	(OA)_2_(FA)_*n*-1_Pb_n_Br_3n+1_	528–532	13.4	34,480	2018	[137]
Oleylamine (OAm^+^)	(OAm)_2_SnBr_4_	625	0.1	350	2019	[138]
Phenylmethylammonium (PMA^+^)	100% PMA + CsPbBr_3_	/	0.69	300	2020	[139]
Phenylethylammonium (PEA^+^)	(PEA)_2_PbCl_2_Br_2_	475	/	70	2018	[140]
	2D PEABr-CsPbBr_3_	513	16.2	31,012	2020	[102]
	PEA_2_SnI_4_	629	0.16	58	2020	[30]
	60%EA: PEA_2_(CsPbBr_3_)_2_PbBr_4_	488	12.1	2191	2020	[141]
	40%EA: PEA_2_(CsPbBr_3_)_2_PbBr_4_	495	13.3	2790	2020	[141]
	80%EA: PEA_2_(CsPbBr_3_)_2_PbBr_4_	480	4.91	83	2020	[141]
	(PEA)_2_(FA)_*n*−1_Pb_n_Br_3n+1_	532	14.36	9120	2018	[142]
	PEA_2_(Rb_x_Cs_1−x_)_2_Pb_3_Br_10_	475	1.35	100.6	2019	[143]
	(PEABr)_x_CsPbBr_3_	514	12.1	10,270	2020	[144]
	PEA_2_(FAPbBr_3_)_2_PbBr_4_	532	15.4	15,765	2019	[145]
	PEA_2_PbBr_4_	410	0.31	147.6	2019	[146]
	P2F8	527	12.4	5200	2019	[147]
	PEA_2_Cs_n-1_Pb_n_Br_3n+1_	518	16.24	30,140	2020	[148]
	CsPbCl_0.9_Br_2.1_ + PEABr	480	5.7	3780	2019	[84]
	PEA_2_Cs_n-_1Pb_n_Br_3n+1_	478	6.3	~ 200	2020	[149]
	CsPbBr_3_: PEACl: YCl_3_	485	11	9040	2019	[120]
	PEA_2_MA_n−1_Pb_n_Br_3n+1_	515.1	9.2 ± 1.43	66,000	2020	[123]
	PEA_2_Cs_1.6_MA_0.4_Pb_3_Br_10_ treated with DPPOCl	479	5.2	468	2019	[150]
Phenylpropylammonium (PPA^+^)	75% PPA + CsPbBr_3_	/	1.87	4700	2020	[139]
Phenylbutylammonium (PBA^+^)	PBA_2_Cs_n-1_Pb_n_I_3n+1_	664	13.3	968	2019	[122]
	PBA_2_(CsPbBr_3_)_*n*−1_PbBr_4_	514	10.4	14,000	2017	[151]
	PBA_2_(CsPbI_3_)_*n*−1_PbBr_4_	683	7.3	/	2017	[151]
	PBABr_y_(Cs_0.7_FA_0.3_PbBr_3_)	483	9.5	54	2019	[152]
2-Phenoxyethylamine (POEA)	POEA(30%): MAPbBr_3_	520	2.82	64.2	2017	[82]
2-Thiopheneethylamine (TEA^+^)	TEA_2_SnI_4_	638	0.62	322	2020	[30]
1-Naphthylmethylammonium (NMA^+^)	(NMA)_2_(FA)_*n*–1_Pb_n_I_3n+1_	795	20.1	/	2018	[44]
	NMA_2_PbBr_4_: Mn(10%)	630	0.004	/	2019	[153]
	NMAI: FABr: PbI_2_ (2: 1.9: 2)	780	12.7	/	2018	[91]
	(NMA)_2_Cs_n−1_PbnI_3n+1_	694	7.3	732	2018	[154]
	60%NMA + CsPbBr_3_	516	6.01	13,200	2020	[139]
	(NMA)_2_PbBr_4_: FABr	514	14.9	2056	2018	[155]
1-Naphthylethylammonium (1-NEA^+^)	60% 1-NEA + CsPbBr_3_	509	2.45	5200	2020	[139]
2-Naphthylethylammonium (2-NEA^+^)	40% 2-NEA + CsPbBr_3_	511	1.98	15,000	2020	[139]
Benzimidazolium (BIZ^+^)	(BIZ)_2_(FA)_*n*−1_Pb_n_Br_3n+1_	535	7.7	30,000	2018	[124]
	(BIZ)_2_Mn_0.23_Pb_0.77_I_4_	575	0.045	225	2019	[121]
3,3-Diphenylpropylammonium (DPPA^+^)	60% DPPACl + CsPbBr_2_Cl	464	1.92	442	2020	[156]
Propane-1,3-diammonium (PDA^2+^)	CsBr + PbBr_2_ + PDABr_2_	490	8.5	2257	2021	[157]
1,4-Bis(aminomethyl)benzene (BAB)	(BAB) FA_n−1_Pb_n_X_3n+1_	776	5.2	/	2019	[79]
PEA^+^ + iso-butylammonium (iso-BA^+^)	(iso-BA_X_PEA_1−X_)_2_Cs_n−1_Pb_n_(Br_0.7_Cl_0.3_)_3n+1_ (x = 4)	485	7.84	1130	2020	[158]
PEA^+^ + iso-propylammonium (IPA)	IPA/PEA_2_MA/Cs_n-1_Pb_n_Br_3n-1_	490	1.5	2480	2018	[118]
PEA^+^ + PA^+^	PEA_x_PA_2−x_(CsPbBr_3_)_*n*−1_PbBr_4_	488	7.51	1765	2020	[159]
PEA^+^ + BA^+^	(BA_0.5_PEA_0.5_)_2_MAPb_2_Br_7_	456	0.015	/	2020	[21]
PEA^+^ + P-PDA^2+^	P-PDABr_2_ + PEABr + CsBr + PbBr_2_	465	2.6	211	2019	[31]
PEA^+^ + dimethylammonium (DMA^+^)	PEA_2_DMA_1.2_Cs_2_Pb_3_Br_11.2_	499	1.58	7760	2020	[160]
PEA^+^ + N-(2-Bromoethyl)-1,3-propanediamine dihydro (NPA^2+^)	PEA + NPA + CsBr + PbBr_2_	485	2.62	1200	2019	[161]
PEA^+^ + DPPA^+^	CsPbCBr_2_ + DPPABr + PEABr	470	8.8	482	2020	[162]
PBA^+^ + PA^+^	(PBA)(PA)A_n-1_Pb_n_X_3n+1_	534	15.1	8052	2020	[45]
PEA^+^ + PA^+^ + ABA^+^	PEA_x_PA_2−x_(CsPbBr_3_)_*n*−1_PbBr_4_ + ABA_2_PbBr_4_	486	10.11	513	2021	[77]

**Fig. 9:**
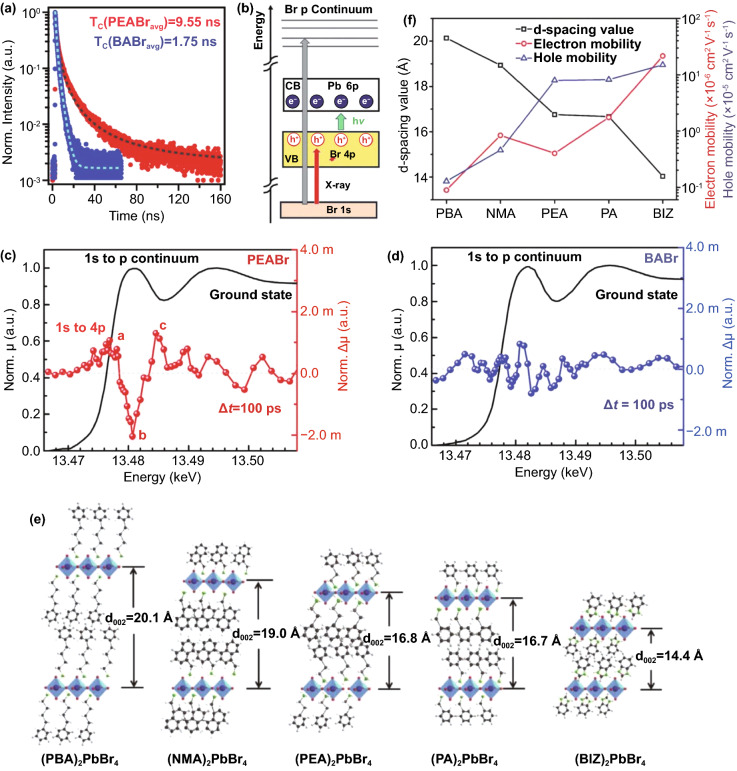
**a** Time-resolved PL decay curves for PEABr and BABr thin films. **b** Schematic illustration of bromide (Br) K near edge transitions. The ground-state X-ray absorption spectra as a function of energy and the change in X-ray absorption after 100 ps laser excitation for **c** PEABr and **d** BABr thin films. Copyright 2020 Wiley–VCH Verlag [123]. **e** Schematic representation of the barrier width of (PBA)_2_PbBr_4_, (NMA)_2_PbBr_4_, (PEA)_2_PbBr_4_, (PA)_2_PbBr_4_ and (BIZ)_2_PbBr_4_ perovskite films. **f** Electron and hole mobilities versus d-spacing value of perovskite films with various organic cations. Copyright 2019 John Wiley and Sons Inc [124]

Jin et al. selected two different organic cations, namely PBA and BA. Through comparison, it was found that different organic amine cations would affect the thicknesses of perovskite quantum wells, efficiency of energy transfer and recombination kinetics, resulting in great differences in the performance of PeLEDs [122]. Similarly, Nie et al. deeply discussed the influence of organic cations with benzyl ring (PEA) or alkyl chain (BA) as spacer on PeLEDs. It is found that the carrier lifetime of PEA-based film is 5 times that of BA-based film (Fig. 9a), which greatly improves the probability of radiative recombination. It is worth noting that the luminance of benzyl ring is 70 times that of alkyl chain, and it has superior output luminance efficiency (about 25 cd A^−1^) and EQE (more than 9%). The results show that such a large difference is closely related to the steric hindrance of organic cations. Compared with alkyl chain, the volume of benzyl ring is larger, which will affect the crystalline packing of inorganic layers. Because the alkyl chain is more flexible, the band structure of perovskite will not be affected. The time-resolved X-ray absorption spectra show that the hole localization signal of benzyl ring film is near the Br *p*-orbital, which increases the carrier lifetime and enhances the radiative recombination efficiency (Fig. 9b–d). Therefore, the PeLEDs of benzyl ring show excellent electroluminescent properties. However, these were not observed in the alkyl chain film [123]. In addition, Wang et al. selected the organic cation BIZ without alkyl branches by comparing the *d*-spacing of various organic cations (Fig. 9e). Because the smaller the *d*-spacing, the shorter the barrier width, which can increase the carrier mobility. Figure 9f shows that BIZ has excellent electron mobility and hole mobility [124]. Using the same method, Pullerits et al. [125] selected iso-BA with smaller *d*-spacing, which significantly increased the transfer rate and reduced the possibility of charge accumulation. Choosing different organic cations will change the intrinsic electronic properties of semiconductors [126], which is helpful to design highly efficient and stable PeLEDs. So far, the choice of spacer cations is not limited to a single organic ion. Various mixed organic cations as spacers have been reported in many excellent works. Choy et al. introduced the bifunctional organic spacer 4-(2-Aminoethyl)benzoic acid (ABA) into PEA_x_PA_2−x_(CsPbBr_3_)_*n*−1_PbBr_4_. Due to the addition of ABA, the interaction between the layers of perovskite was enhanced, and Pb was suppressed to reduce the trapped states and promote the reduction of non-radiative recombination loss. The blue 2D PeLEDs with good performance (EQE = 8.21%) and high stability (*T*_50_ = 81.3 min) were obtained [77]. From the perspective of theoretical calculation, taking *n* = 2 as an example, the performance of mixed organic cation LEDs is better than that of single spacer cation LEDs, which is attributed to lower formation energy, lower strain and lower electron–phonon coupling [21].

In a word, the chain length, size and properties of organic cations may affect the performance of the device. Compared with alkyl chain, organic cations with large volumes are selected, such as traditional PEA^+^ and PBA^+^. The higher the molecular packing density in the organic layer is, the higher the rigidity of the crystal will be. Sargent et al. showed that 2D perovskite single crystals with high PLQY and good quality can be obtained by controlling the crystal rigidity [74]. In addition, the selection of organic cations with small spacing also helps to increase PLQY, which is due to the decrease of the barrier width of charge transport and the increase of carrier mobility.

#### Adjusting Width of Quantum Well

**Fig. 10:**
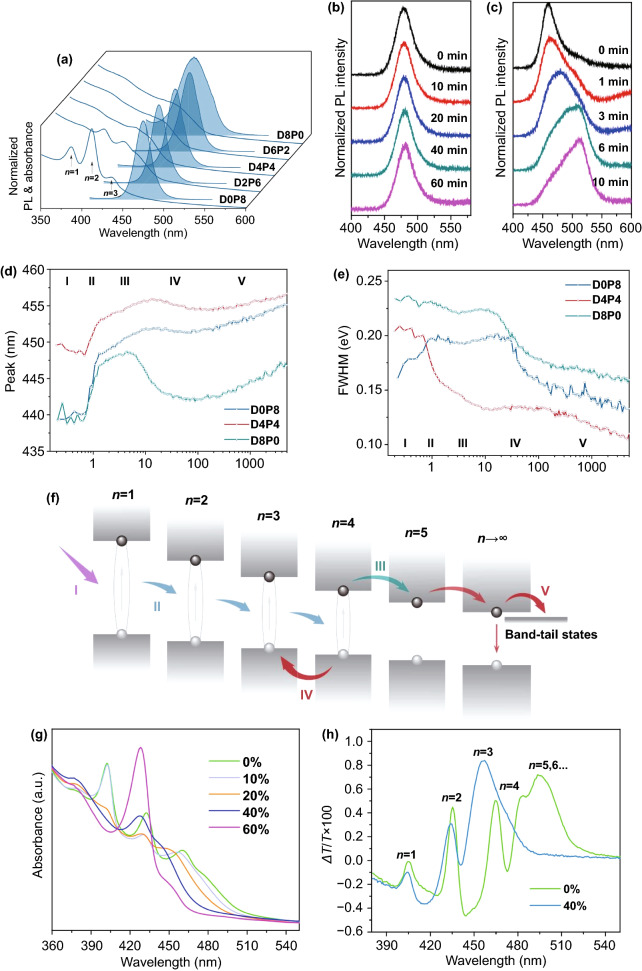
**a** Steady-state PL and absorption spectra. PL spectra of **b** PEA_2_A_1.5_Pb_2.5_Br_8.5_ with 40% IPABr and **c** MAPbCl_1.5_Br_1.5_ under continuous laser radiation for different exposure times. The evolution of **d** peak and **e** FWHM are extracted from the broad bleach peak (425–470 nm) of samples D0P8, D4P4 and D8P0. **f** Schematic diagram of carrier behaviors after excitation. I, carrier formation; II, exciton transfer; III, charge transfer; IV, reverse charge transfer; V, continuous charge transfer and recombination. **g** Absorption spectra of perovskite PEA_2_A_1.5_Pb_2.5_Br_8.5_ with 0 − 60% IPABr. **h** TA spectra of PEA_2_A_1.5_Pb_2.5_Br_8.5_ with 0 and 40% IPABr. Copyright 2018 Springer Nature [118]. Copyright 2020 Springer Nature [162]

As we known, effective energy transfer plays a decisive role in the photoluminescence efficiency of 2D perovskites. Reasonable distribution of quantum well width can reduce unnecessary energy loss and facilitate carrier transport. To achieve high-performance PeLEDs, it is necessary to study the influence of quantum well width distribution on devices. Zhong et al. controlled the *n* values of perovskite films by adjusting the ratio of DPPABr to PEABr. Figure 10a shows that the larger the DPPABr/PEABr ratio is, the larger the *n* domains are. Interestingly, there are multiple emissions when DPPABr content is the highest. DPPABr promotes the formation of large *n* domains, while PEABr is beneficial to the formation of small *n* domains. DPPABr and PEABr are mixed in a certain proportion to realize the regulation of quantum well width distribution. Furthermore, the carrier dynamics process is divided into five stages as shown in Fig. 10f. Firstly, photogenerated carriers will be formed in the 2D perovskites after photoexcitation, resulting in the increase of bleach peak signal. The peak and FWHM fluctuations in Fig. 10d, e are due to the competition of different *n* values bleach signals. Then, the carriers transfer rapidly (0.7–1.3 ps) and the bleach peak shifts red. In the third stage, the excitons are decomposed into free carriers and charge transfer occurs, resulting further red shifts of the bleach peak. During the whole process, the FWHM of D4P4 decreases most rapidly (Fig. 10e), indicating the most effective energy transfer and the narrowest distribution of quantum well width. In the fourth stage, due to the blueshift of the bleach peak (about 100 ps) caused by the reverse charge transfer, the energy cannot be concentrated in the effective emission region. Lastly, the free carriers are transferred to the layer with large *n* values or band-tail states and recombined through radiative or non-radiative processes. The combination of DPPABr and PEABr not only reduces the small *n* value which is not conducive to the emission efficiency, but also shortens the width of quantum well. Likewise, Sargent et al. used IPA and PEA to control the distribution of quantum well width by combining various cations. Their result indicated that the absorption edge shifts blue after inducing IPABr, indicating that the formation of small *n* value phases and large *n* value phases is inhibited (Fig. 10g). And Fig. 10h also shows that the existence of large *n* phases (*n* = 5, 6…) is not observed after adding 40% IPABr to PEA2A1.5Pb2.5Br8.5. In addition, the stability of the device is also enhanced (Fig. 10b, c).

#### Management of Singlet and Triplet Excitons

**Fig. 11:**
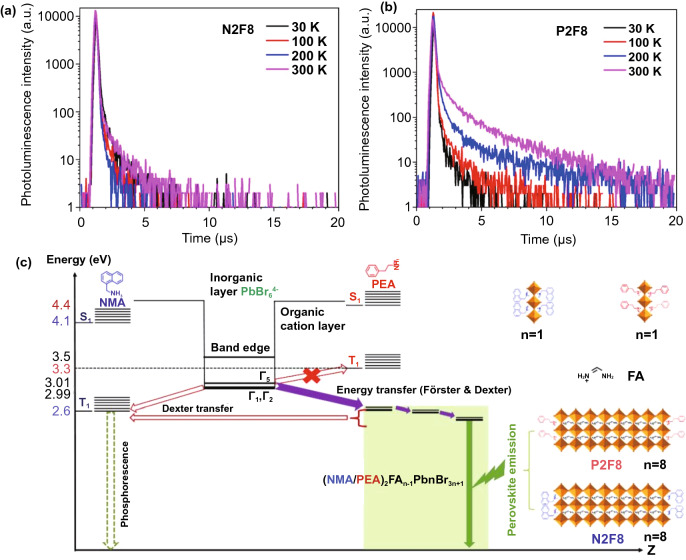
Photoluminescence decay curves of **a** N2F8 and **b** P2F8. **c** Energy transfer mechanism of quasi-2D perovskites under light excitation. Where S is the singlet state and is the triplet states. Copyright 2019 Springer Nature [147]

The management of triplet excitons plays a key role in the design of efficient and stable organic OLEDs. Although the influence of excitons on the performance of PeLEDs is not clear, Adachi et al. used the management strategy of triplet excitons and selected organic cation NMA^+^ as the comparison of PEA^+^, which proved the importance of triplet excitons on 2D PeLEDs. It can be seen from Fig. 11a, b that the PL decay curves of (NMA)_2_ FA_7_Pb_8_Br_25_ (N2F8) and (PEA)_2_FA_7_Pb_8_Br_25_ (P2F8) are obviously different. The decay curve of N2F8 hardly changes under different temperature. However, P2F8 shows fast and slow decay with the increase of temperature, which is due to the thermally activated reverse intersystem crossing (RISC) of triplet excitons. Excitons are formed in 2D perovskites by light excitation. The singlet states transfer rapidly to the large *n* domains, but the triplet states are different. As shown in Fig. 11c, PEA^+^ has a higher triplet energy level than NMA^+^. And the lowest excited triplet T_1_ of PEA^+^ is 3.3 eV, which is higher than all triplet energy levels, so PEA^+^ can effectively obtain triplet excitons. However, the T_1_ of NMA^+^ is 2.6 eV, which is lower than the triplet exciton levels Γ_1_ and Γ_2_ of [PbBr_6_]^4−^, leading to Dexter energy transfer. Dexter energy transfer will compete with energy transfer in 2D perovskites. The high-efficiency PeLEDs with EQE and current efficiency of 12.4% and 52.1 cd A^−1^ are rationally designed by the effectively management of the triplet states [147].

### Enhanced Energy Transfer

**Fig. 12:**
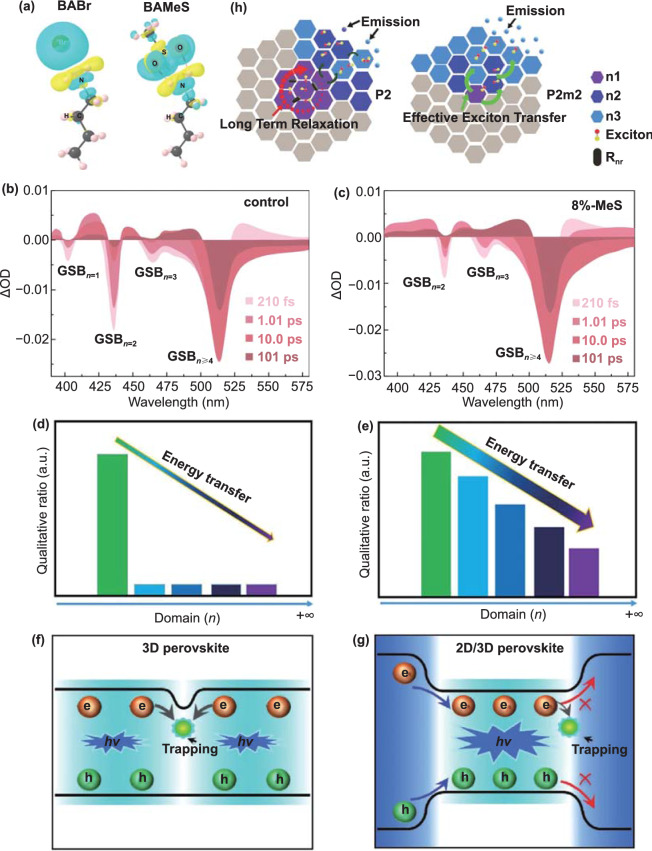
**a** Hydrogen bond calculations. Differential charge density diagrams of BABr and BAMeS (where isosurface value is 0.0015 eV Å^−3^; cyan is charge accumulation; yellow is charge depletion). TA spectra at selected timescales of the **b** control and **c** 8%-MES perovskite films. Copyright 2021 Springer Nature [134]. The energy transfer process of **d** BAB33 and **e** BAB25 perovskite films was studied. Copyright 2019 American Association for the Advancement of Science [79]. Comparison of **f** 3D and **g** 2D/3D perovskite film carrier recombination. Copyright 2020 Wiley–VCH Verlag [163]. **h** Domain distribution, exciton transfer and emission properties of P2 and P2m2 films. Copyright 2019 Wiley-Blackwell [31]

As mentioned above, compared with 3D perovskite, 2D perovskite has a larger *E*_*b*_, and the electrons and holes are effectively confined to enhance the radiation recombination [142]. Moreover, 2D perovskite structure is a common method to construct high-performance PeLEDs. However, due to the random stacking of 2D perovskites, the distribution of *n* domains is uneven and multiple emission peaks appear. In addition, small *n* phases are formed first in 2D perovskites because of the low formation energy of small *n* phases [158]. All of these have negative effects on the energy transfer from small *n* domains to large *n* ones. In particular, the radiation of *n* = 1 phase belongs to non-radiation recombination, which seriously affects the performance of PeLEDs. Yang et al. introduced methanesulfonate (MES) to reconstruct the phase distribution of perovskite and promote the energy transfer from small *n* phase to large *n* one. The density functional theory (DFT) calculation shows that SO_3_^−^ in MES tends to form a strong force with organic cation BA^+^, and the charge redistribution between them is 0.84 e (electron), which is greater than that between BA^+^ and Br^−^ (0.70 e) (Fig. 12a). The existence of hydrogen bonds increases large *n* domains, which can regulate the crystallization kinetics. In Fig. 12b, the exciton resonance at GSB_n=2_ still exists after long-time excitation at 101 ps. However, the exciton resonance at GSB_n=2_ is not found in the 8%-MES films (Fig. 12c). The reason resulting in the noticeable difference is due to the effect of effective energy transfer. It is gratifying that the exciton resonance of GSB_n = 1_ is not found in the 8%-MES films. The introduction of MES successfully inhibits the formation of small *n* phases. Eventually, quasi-2D green PeLED with EQE up to 20.5% is obtained [134]. Ning et al. achieved high carrier transfer efficiency based on DJ structure by adjusting the ratio of BAB to FA in (BAB)FA_n−1_Pb_n_X_3n+1_ (*X* = Br, I). As shown in Fig. 12d, e, the peak intensity of BAB25 in the large *n* domain is about twice that of BAB33 [79]. As we all known, many defects in 3D perovskites are not conducive to radiative recombination (Fig. 12f). Zeng et al. used the 2D/3D perovskite structure to make the energy levels connected and enhance the energy transfer. The formation of energy level cascade channels makes the energy transfer from the wide bandgap to the narrow bandgap (Fig. 12g), which enhances the radiative recombination efficiency. Although the organic ligands of 2D perovskites do not contribute to the charge transfer, the free charge diffusion is inhibited in 3D perovskites, which can just combine with the opposite charge. The coordination effect of the above two factors has successfully increased the EQE value by about 5 times, and the stability has also been greatly improved [163]. By introducing [1,4-Bis(aminomethyl)benzene bromide (P-PDABr_2_)], the exciton shifts rapidly from small *n* domains to large ones (Fig. 12h).

## Current Challenges Ahead in 2D PeLEDs

### Improving Efficiency of Blue 2D PeLEDs

**Fig. 13:**
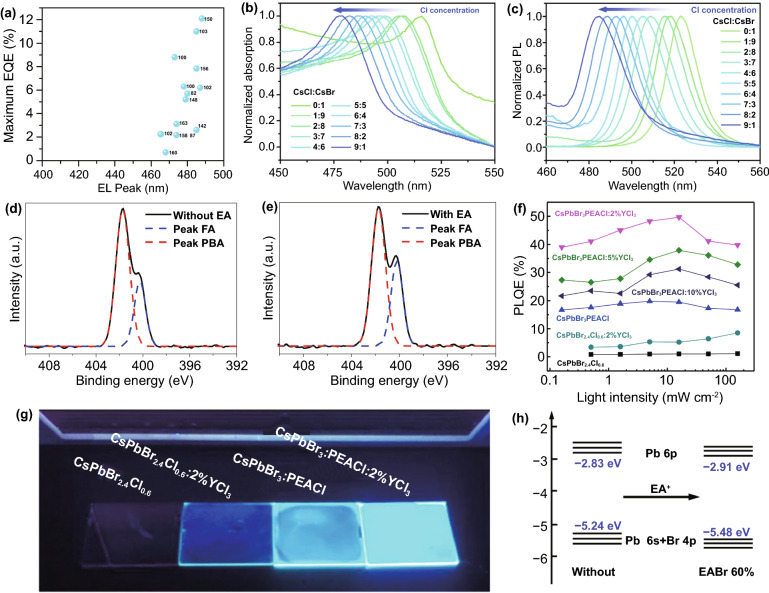
**a** Comparison of EQE values of 2D perovskites blue LEDs with EL peak in the range of 400–500 nm. **b** Normalized absorption and **c** photoluminescence spectra of CsPbClxBr3 − x thin films with different molar ratios of CsCl to CsBr. Copyright 2019 Springer Nature [84]. High-resolution N 1s XPS spectra of the perovskite films without **d** and **e** with EA treatment. Copyright 2019 Springer Nature [152]. **f** A photograph of the perovskite films under the ultraviolet lamp. **g** Power-dependent PLQEs of films with different compositions. Copyright 2019 Springer Nature [120]. **h** Diagram of energy levels change of quasi-2D perovskite in Pb 6p and (Pb 6 s + Br 4p) orbitals with EA + insertion. Copyright 2020 Springer Nature [141]

Since 2014, the largest EQEs of green and red PeLEDs have exceeded 20%, and have made great achievements [164–170]. However, as one of the primary colors of high-definition displays [171], the development of blue PeLEDs is quite slow, far behind red and green PeLEDs. As shown in Fig. 13a. The universal strategy for preparing blue emission is to control the mixed halogen anions so that achieve tuning of continuous bandgap by adding chlorine (Cl) to the bromide (Br)-based perovskites and modulating the proportion of halogen ions (Fig. 13b, c) [84, 172]. But the spectral shift of these mixed perovskites usually occurs under different operating voltages, which is due to the migration and segregation of mixed halides [173, 174]. In addition, different metal ions such as Mn^2+^, Al^3+^, Cu^2+^ and Zn^2+^ are doped into the B site to adjust the conduction band [175–178]. Unfortunately, the impurity energy levels induced by metal ions are disadvantageous to radiative recombination. At present, it is also an important strategy to achieve blue emission by inserting large organic cations into 3D perovskites. Ma and co-workers not only introduced PEABr into CsPbX_3_, but also rearranged the 2D perovskite phase distribution with NaBr, which reduced the formation of a small *n* domain (*n* = 1) dominated by non-radiative recombination and increased PLQY from 39 to 67%. The sky-blue PeLED with EQE up to 11.7% was obtained [78]. However, the charge transport capacity of 2D perovskites is poor due to the organic compounds acting as spacer cations. Jin et al. used ethyl acetate (EA) as the anti-solvent, which was beneficial to the dissolution of PBABr, and would not damage the emission layer and the underlying layer. As shown in the high-resolution *N* 1s XPS spectra, EA changed the ratio of PBA^+^ and FA^+^ (Fig. 13d, e) [152]. Huang et al. introduced PEACl into CsPbBr_3_ to facilitate the formation of layered perovskites, and further doped YCl_3_, resulting in a qualitative leap in the PLQE of the films, from 1.1 to 49.7% (Fig. 13f). CsPbBr_3_: PEACl: 2% YCl_3_ exhibits the brightest luminescence under the ultraviolet lamp (Fig. 13g). However, the existence of yttrium on CsPbBr_3_ grain increases the bandgap and forces the radiative recombination of carriers in CsPbBr_3_. The sky-blue PeLED with a maximum brightness of 9040 cd m^−2^ and EQE of 11.0% are prepared [120]. The above efficient perovskite blue LEDs mainly focus on the modification of organic cations. You et al. used EA^+^ to replace Cs in PEA_2_(CsPbBr_3_)_2_PbBr_4_ at A site, which makes the maximum valence band of the 2D perovskite moves down and the bandgap increase (Fig. 13h). So far, this may be the highest efficiency of sky-blue LED (12.1%). Moreover, the device shows excellent stability and can survive for as long as 1 h without encapsulation in nitrogen [141]. Although 2D perovskite blue LEDs have made great progress, their applications in full-color display and white lighting are still far away, which is a huge challenge.

### Enhanced Stability of 2D PeLEDs

At present, some excellent works have reported more than the EQEs of 2D PeLEDs exceed 20%. Unfortunately, the stability of 2D PeLEDs is poor, which is a stumbling block in practical application. As we all know, T_50_ is one of the important parameters to evaluate the stability of LEDs. However, at the low luminance of 100 cd m^−2^, the T_50_ values of most blue 2D PeLEDs reported so far are tens of minutes [120, 152, 161], some even tens of seconds [78, 141], which are far from those of OLED and QD LEDs. Compared with perovskite solar cells, PeLEDs need to work in higher applied electric field. Ion migration is the main factor leading to poor stability of 2D PeLEDs. To enhance the stability of PeLEDs, it is necessary to effectively suppress ion migration. Because ion migration mainly occurs at grain boundaries, preventing ion migration along grain boundaries is a feasible way to suppress ion migration. Lee et al. designed proton-transfer-induced 2D perovskite formation while retaining 3D perovskite. Compared with MA^+^, due to the π − π interaction and steric repulsion of Benzylamine cation (BnA^+^), the reorientation rate is significantly reduced and the ion migration along grain boundaries is effectively suppressed [179]. Furthermore, the decomposition of organic cations a voltage bias is one of the reasons for the instability of 2D PeLEDs. Snaith et al. found that phenylethylammonium bromide (PEABr) greatly improves the efficiency of CsPbBr_3_ LEDs, but PEABr will decompose and produce mobile ions, which will move to ETL, eventually leading to the charge imbalance of the device [144]. In RP phase, the adjacent inorganic octahedral slabs have an inherent dissociation tendency, which also leads to the decomposition of perovskites, and the organic cations are connected by weak van der Waals force. Therefore, enhancing the interaction between large organic cations and octahedral slabs is beneficial to improve the stability of 2D PeLEDs. The octahedral slabs of DJ phase are bridged by diamine cations, and the interaction force is stronger than that of monoamine cations of RP phase [157]. Ning et al. used 1,4-bis(aminomethyl)benzene (BAB) as bridging molecules to obtain a 2D PeLED with *T*_50_ lifetime up to 100 h, which is almost two orders of magnitude longer than that of RP phase (PEA as organic spacer cation) [79].

In addition, the stability of PeLEDs is also related to the thermal stability of the material. Under the continuous operating voltage, the Joule heat causes the perovskite interface to be heated, leading to degradation. Most of the 2D perovskite films are easy to transform into mixed 2D/3D phases when heated [180]. Moreover, the efficiency of the devices will be adversely affected if the annealing temperature is too high and the annealing time is too long [181].

### Toxicity Reduction of 2D PeLEDs

Most of the researches on 2D perovskite optoelectronic devices are mainly based on lead-based perovskites at the moment. Although the lead content meets the standard of commercial products, lead-based perovskite is easily soluble in water. If the encapsulant material is damaged, lead leakage occurs and seeps into the water, which will cause serious pollution to the environment. In recent years, lead-free tin-based perovskite has attracted people's attention [31, 182]. Unfortunately, Sn^2+^ is easily oxidized to Sn^4+^. Moreover, the crystallization rate of Sn-based perovskite is faster than that of Pb-based perovskite, resulting in more defects. The films are prone to non-radiative recombination caused by defects, which seriously affects the performance of devices. Sargent et al. introduced liquid reductant H_3_PO_2_ into the precursor solution to inhibit the oxidation of Sn^2+^, and obtained red LED with maximum brightness of 70 cd m^−2^ and EQE of 0.3% [183]. Soon after, they developed a new strategy, using valeric acid to slow down the Sn-based crystallization rate and avoid the formation of Sn^4+^. Thus, the lead-free red PeLED with the longest half-life and the highest EQE (5%) was obtained [114]. Dou et al. used 7-(thiophen-2-yl)benzothiadiazol-4-yl)-[2,2’bithiophen]-5-yl)ethylammonium iodide (BTmI) as the barrier layers to display a pure red Sn-based PeLED with a luminance of 3466 cd m^−2^ and a working stability of more than 150 h [184]. (111)-oriented 2D perovskites have also been used as LEDs, but they all show unsatisfactory efficiency. The first example using Cs_3_Sb_2_I_9_ as the emitting layer was reported by Chu et al. The wavelength of Cs_3_Sb_2_I_9_ is controlled by the vapor anion exchange method. The device shows an average radiance of 0.012 W sr^–1^ m^–2^ at 6 V [185]. Shan and his colleagues reported lead-free Cs_3_Sb_2_Br_9_ with EQE of ∼0.206%, which is the shortest wavelength PeLED known to us [186]. Even though the performances of lead-free PeLEDs are far from that of lead-based perovskite, there is a great room for development.

## Conclusions and Outlook

In this review, we summarize the applications of 2D perovskites in LEDs in recent years, mainly RP perovskites. Compared with typical 3D ABX_3_, 2D perovskites have natural quantum well structures, larger E_b_ and better ambient stability. The structure of 2D perovskites can be regarded as the periodic splitting of 3D perovskites along (100), (110) and (111) planes by organic cations. RP perovskites belong to (100)-oriented 2D perovskites. Here, we analyze and compare the structures of 2D perovskites with different orientations. In this direction, more and more new members have been found, increasing members of halide perovskite group. Moreover, 2D perovskites have many excellent properties, such as convenient control of the width of quantum wells, formation of dense films and ultra-fast energy transfer, which shows great potential in the field of optoelectronic devices. However, there are many types of organic spacers, so how choose organic cations correctly is the key to promote the development of PeLEDs. The effects of steric hindrance and alkyl chain length on the performance of the devices are briefly described. Although many alkylamines or aromatic amines have been explored as spacers, there is still a lot of space to expand the 2D perovskites family, which is helpful to understand the 2D perovskites comprehensively and to grasp the influence of perovskites structures and properties on device performance fundamentally.

Unfortunately, the introduction of organic cations also has negative effects. Due to the rapid nucleation, 2D perovskites usually contain many different *n* values, resulting in multipeak emission, which is fatal for high color purity display. Therefore, the accurate control of phase purity is one of the key factors in the design of efficient and stable PeLEDs. Although some promising results have been achieved, there is still a long way to go for large-scale productions of films. It is necessary to further study the nucleation and growth mechanism of 2D perovskites. In addition, as an emerging technology, the stability of 2D PeLEDs is also a big obstacle to their practical applications. In 2D perovskite materials, although large organic cations can improve the humidity resistance of films, the stability of devices is still threatened by ion migration, thermal instability and interface instability. Currently, passivation of surface defects and control of crystal growth rate is often used to alleviate the instability of perovskites phase. How to obtain long-term stable and efficient 2D PeLEDs needs further research.

In summary, due to the unique photoelectric performance of quasi-2D, PeLEDs have experienced a blowout type development in just seven years. It has the potential to surpass OLEDs and QD LEDs and is expected to be used in next-generation displays and lighting equipment. Although remarkable achievements have been made, many problems still remain unsolved. We hope that this review will provide a comprehensive summary for society to deepen the understanding of 2D PeLEDs.
